# Suspected and Confirmed Vector-Borne Rickettsioses of North America Associated with Human Diseases

**DOI:** 10.3390/tropicalmed3010002

**Published:** 2018-01-03

**Authors:** Melissa Hardstone Yoshimizu, Sarah A. Billeter

**Affiliations:** 1 Vector-Borne Disease Section, California Department of Public Health, 850 Marina Bay Parkway, Richmond, CA 94804, USA; melissa.yoshimizu@cdph.ca.gov; 2 Vector-Borne Disease Section, California Department of Public Health, 2151 Convention Center Way Suite 218B, Ontario, CA 91764, USA

**Keywords:** arthropods, fleas, mites, North America, *Rickettsia*, rickettiosis, ticks

## Abstract

The identification of pathogenic rickettsial agents has expanded over the last two decades. In North America, the majority of human cases are caused by tick-borne rickettsioses but rickettsiae transmitted by lice, fleas, mites and other arthropods are also responsible for clinical disease. Symptoms are generally nonspecific or mimic other infectious diseases; therefore, diagnosis and treatment may be delayed. While infection with most rickettsioses is relatively mild, delayed diagnosis and treatment may lead to increased morbidity and mortality. This review will discuss the ecology, epidemiology and public health importance of suspected and confirmed vector-transmitted *Rickettsia* species of North America associated with human diseases.

## 1. Introduction

*Rickettsia* (Order: Rickettsiales, Family: Rickettsiaceae) are Gram-negative, obligate, intracellular alphabacteria; more than 30 species and subspecies are reported worldwide and more than half are confirmed or suspected human pathogens [1]. Over the past two decades, identification of rickettsial agents pathogenic to humans and/or animals has greatly expanded [2,3]. Typically, rickettsiae are transmitted to vertebrate hosts via a hematophagous arthropod vector such as ticks, fleas, or mites. Rickettsiae are transmitted by the arthropod vector either directly through an infectious bite or via inoculation of infectious fecal material [4]. Once in the bloodstream of a vertebrate host, rickettsiae invade endothelial cells of the vasculature ultimately leading to detachment and death of the infected cells [5]. Rickettsiae will subsequently invade new endothelial cells and can be ingested by a hematophagous arthropod during a blood meal. Once inside the arthropod, rickettsiae will infect and replicate within the epithelial cells lining the midgut, circulate in the hemolymph [6] and may invade the ovaries, salivary glands and other tissues [7,8]. Many rickettsiae are maintained in the arthropod vector by transovarial (female-to-progeny) and/or transstadial (one life stage to the next) transmission [8]. Possible mechanisms utilized by some rickettsial species as a way to infect naïve arthropod vectors while feeding on a non-rickettsemic host include co-feeding and sexual transmission routes [9,10]. 

In North America, the majority of reported human rickettsiosis cases are associated with ticks but rickettsiae transmitted by fleas, lice, mites and other arthropods are also responsible for clinical disease (Table 1). Signs and symptoms associated with rickettsial infections are nonspecific or mimic other infectious diseases [11]; therefore, diagnosis and treatment may be delayed or ineffective. Patients often present with ’flu-like symptoms such as fever, headache, myalgia, lymphadenopathy, rash and/or eschar (*tache noire*) [1]. Doxycycline is the recommended treatment for all rickettsioses and mortality is decreased for patients receiving appropriate antibiotics early in the course of disease [5]. Without appropriate treatment, mortality may reach as high as 20%, particularly with infections of *Rickettsia rickettsii*, the agent of Rocky Mountain spotted fever [12]. Physician education and limiting exposure to hematophagous arthropods are imperative steps to prevent future transmission or severity of infection. This review will discuss the ecology, epidemiology and public health importance of suspected and confirmed vector transmitted *Rickettsia* species of North America associated with human diseases. 

## 2. Tick-Borne Rickettsiae (in Alphabetical Order)

### 2.1. Rickettsia amblyommatis

*Rickettsia amblyommatis* (formerly *Candidatus* Rickettsia amblyommii) [13] was isolated in 1973 from an adult *Amblyomma americanum* tick from Tennessee. Pathogenicity descriptions of human infection with this bacterium are limited [14]. In 2006, a patient from North Carolina developed a macular rash at the site of an attached and partially engorged *A. americanum* tick [15]. The patient had no other symptoms or known laboratory testing but the rash cleared after treatment with doxycycline. The attached tick was tested using PCR and the *ompA* sequence analysis was 100% identical to *R. amblyommatis*. In the southeastern and central United States, the etiologic agent responsible for the condition known as southern tick-associated rash illness (STARI) is unknown. STARI is directly associated with the bite of *A. americanum* and usually includes an expanding rash within seven days of tick bite, fever, headache, fatigue and muscle pain (https://www.cdc.gov/stari/disease/index.html). Retrospective serologic surveys of patients from southeastern and midwestern United States did not implicate *R. amblyommatis* as the causative agent of STARI [16] but have indicated its association with clinical illness [17,18]. Patients with reactivity to *R. amblyommatis* by IFA and Western blot most commonly exhibited fever, headache and myalgia, followed less commonly by rash and thrombocytopenia [18]. 

A survey of reported human RMSF cases and local *A. americanum* ticks conducted in North Carolina indicated that 44% (11/25) of *A. americanum* tick pools were PCR positive and three of six probable RMSF cases demonstrated a fourfold IgG titer rise to *R. amblyommatis* antigens but not *Rickettsia rickettsii* antigens [19]. *Amblyomma americanum* surveys in the United States detected infection in the larval stage, indicating the occurrence of transovarial transmission of *R. amblyommatis* [20,21]. Adult tick infection prevalence ranges from 18% to 44% [22,23,24] and nymphal infections with *R. amblyommatis* range between 3% and 67% [22,23]. *Amblyomma americanum* is a ubiquitous and aggressive human-biting tick primarily distributed in the southern region of the United States but has expanded its range into the northeastern states [25]. Although the geographic distribution of *A. americanum* seems to correlate most closely to human exposure [26,27] and infections with *R. amblyommatis* [28], *R. amblyommatis* can be detected in *Amblyomma* spp. ticks in other parts of North America. In Costa Rica, *R. amblyommatis* was isolated from *Amblyomma cajennense* ticks [29] and in Mexico *Amblyomma mixtum* and *A. cajenennse* have tested positive by PCR [30,31]. 

### 2.2. Rickettsia massiliae

*Rickettsia massiliae* was first isolated in 1985 from an infected patient from Sicily, Italy and was identified from blood-inoculated Vero cells two decades later in 2005. The patient was hospitalized with fever, maculopapular rash on the palms and soles, and an eschar [32]. A case with similar clinical presentation described in 2005 from Argentina was confirmed as *R. massiliae* by PCR testing of the eschar biopsy [33]. The eschar presentation and geographic overlap of *R. massiliae* and *Rickettsia conorii* (etiologic agent of Mediterranean spotted fever) may result in the underdiagnosis and underreporting of *R. massiliae* infections. 

Multiple genotypes of *R. massiliae* have been detected in several species of the *Rhipicephalus* spp. complex from Europe [34], Africa [35] and the United States [36,37,38]. *Rhipicephalus sanguineus*—the brown dog tick—is distributed throughout the world due to its anthropophilic nature and its affinity for feeding on domestic dogs. Generally, *R. sanguineus* has low affinity for humans but in high infestations and with the proper climatic conditions, immature stages of *R. sanguineus* can exhibit increased biting rates of humans [39]. *Rickettsia massiliae* strain AZT80 has been isolated from *R. sanguineus* ticks in Arizona, California and Virginia [36,37,38]. Phylogenetics, using multiple loci, showed that these North American isolates shared the most sequence identity with the Bar29 strain—with unconfirmed pathogenicity—isolated from Spain [38]. While no human cases have been reported from North America, the confirmed case reported from Argentina highlights the potential for broader geographic distribution of this potentially disease-causing pathogen in the Americas where the pathogen and primary vector, *R. sanguineus*, are found. In some urban areas, infestations of *R. sanguineus* are high and canine sickness from *R. massiliae* may be present [37]. 

### 2.3. Rickettsia montanensis

*Rickettsia montanensis* was implicated as the etiologic agent in a single rickettsiosis case in 2011 from Georgia [40]. The juvenile patient developed a nonpuritic, bilateral rash on the lower body—including a maculopapular rash on the soles—four days after a tick was removed. The patient further developed headache and nausea symptoms. The removed tick identified as *Dermacentor variabilis* was PCR positive for *R. montanensis* [40]. Despite supportive clinical data, this patient did not meet the national surveillance case definition for infection with spotted fever group rickettsia, due to lack of fever. However, the patient did meet the laboratory criteria for confirmed diagnosis [40,41]. Infection studies show that dogs remain healthy when exposed to *R. montanensis* via intradermal inoculation [42] or natural exposure to infected ticks [43]. While *R. montanensis* generally is regarded as non-pathogenic, the human case suggests potential pathogenicity.

Tick surveillance in Georgia detected *R. montanensis* in *D. variabilis* and *A. americanum*. Moreover, a survey of ticks found attached to Georgia residents demonstrated 10% of *D. variabilis* and 0.4% of *A. americanum* were PCR positive for *R. montanensis* [14]. Surveys of questing ticks of both species in other parts of the eastern United States identified infection prevalence ranging from 2% to 10% in *D. variabilis* and 0% to 0.4% in *A. americanum* [22,23,44]. Surveillance in areas with sympatric *D. variabilis* and *A. americanum* have found that *D. variabilis* generally has a higher infection prevalence of *R. montanensis* [45,46,47] and is often the lone species of *Rickettsia* detected. Modeling based on distribution of *R. montanensis*-positive *D. variabilis* collected off United States military personnel suggest that the highest probability of human infection occurs in the upper Midwest and mid-Atlantic United States [48]. 

### 2.4. Rickettsia parkeri

*Rickettsia parkeri* was first isolated in 1937 from an *Amblyomma maculatum* tick collected in Texas, and in a guinea pig animal model, *R. parkeri* caused disease [49]. It was generally regarded as non-pathogenic to humans until it was associated with human disease in 2002 [50]. The index patient from Virginia presented with fever, headache, malaise, myalgia and arthralgia, multiple eschars and later developed a nonpruritic, erythematous maculopapular rash on the body, palms and soles. The patient was exposed to ticks frequently through contact with domestic pets but did not recall a specific tick bite. Bacterial cells were observed in the immunohistochemical evaluation of the eschar skin biopsy. DNA sequences derived from the eschar biopsy (culture and PCR) yielded a genetic match to *R. parkeri* [50]. In 2006, a second documented human case was reported from a patient who had returned from Virginia, presenting with fever, malaise, rash and eschar at the site of a previously-attached tick [51]. *Rickettsia parkeri* was isolated in cell culture from a biopsy of the eschar. Confirmed cases have since been reported from Arizona, Georgia, Kentucky, Maryland, Mississippi, South Carolina, Texas and Virginia [52,53,54,55]. 

In the United States, *R. parkeri* is most commonly detected in *A. maculatum* ticks. *Amblyomma maculatum* is found throughout the Americas. In North America, its range is as far south as Costa Rica and northward into the southeastern region of the United States. *Rickettsia parkeri* has been detected in *A. maculatum* ticks from Florida, Georgia, Kentucky, Mississippi, North Carolina, Oklahoma, South Carolina, Tennessee and Virginia [22,44,56,57,58,59] with infection prevalence typically around 15%. Particularly high rates of *R. parkeri* infection (42–56%) in questing adult *A. maculatum* were detected in southeastern Virginia [22]. One confirmed and one probable case of *R. parkeri* rickettsiosis were acquired in southern Arizona and were likely vectored by *Amblyomma triste* ticks [60]; three ticks associated with this case cluster were PCR positive for *R. parkeri* [54]. 

Domestic dogs do not appear to exhibit clinical symptoms when infected with *R. parkeri*; it is unknown if they become rickettsemic [61]. In a study of shelter dogs in southern Louisiana, 13% of dogs were PCR positive for *R. parkeri*, although blood cultures were negative or unsuccessful [61]. Less than 25% of wild caught small mammals and northern bobwhite quail (*Colinus virginianus*) showed serological evidence of exposure to *R. parkeri* [62]. Laboratory investigations examining the infectivity of *R. parkeri* to cotton rats and northern bobwhite quail, vertebrate hosts for larval and nymphal stages of *A. maculatum*, show that infection is cleared quickly from both vertebrate species; therefore, they are not likely reservoirs [63] because the duration of rickettsemia is not long enough for a tick to acquire infection. Field-caught *A. americanum*, *D. variabilis*, *R. sanguineus* and *Haemaphysalis leporispalustris* have on occasion also tested positive for *R. parkeri* and transstadial and transovarial transmission of *R. parkeri* were demonstrated in *A. americanum* in laboratory studies [64,65]. However, these tick species are not typically associated with transmission of this pathogen [65,66]. 

### 2.5. Rickettsia philipii

*Rickettsia philipii* (prototype strain 364D) was originally isolated in 1966 from *Dermacentor occidentalis* collected in Ventura County, California [67]. Isolates originating from *D. occidentalis* collected in Humboldt and Monterey counties, California, were similar to the 364D prototype strain and could produce clinical manifestations in guinea pig models and in chick embryo infection studies [68]. *Rickettsia philipii* was demonstrated to cause human disease in 2008 with an initial human case reported in rural northern California [69]. The elderly patient was afebrile and the only clinical manifestation was an eschar with swelling and erythema. Molecular diagnostic testing of the eschar fixed tissue biopsy was identical to strain 364D. The patient did not recall tick bite. Since the patient reported no travel history, environmental tick surveillance around the residence was conducted and 16% of *D. occidentalis* ticks were positive for *R. philipii* [69]. Pacific Coast tick fever (PCTF), the disease associated with *R. philipii* infection, has since been reported in 14 patients from California [69,70,71]. Three patients were aware of tick bites prior to illness onset. While four patients required hospitalization, clinical manifestations are characterized primarily by eschar, fever, headache and lymphadenopathy, with rash being uncommon [70]. The majority of these cases have occurred in northern California with a single case reported from southern California [71]. 

*Rickettsia philipii* has been detected in *D. occidentalis* ticks only [68]. This tick species is distributed throughout California and into southern Oregon and northern Baja California, Mexico [72]. *Dermacentor occidentalis* ticks have tested positive from 15 California counties, with average statewide infection prevalence of 2% in adults, 0.9% in nymphs and a minimum infection prevalence of 0.4% in larval pools [71]. Tick surveillance results of *R. philipii* in *D. occidentalis* collected from five southern California counties indicated higher prevalence in this region, with an average infection of 7% [73,74]. Acarological and epidemiological surveillance lends evidence towards nymphal *D. occidentalis* as the primary vector of *R. philipii* to humans, with infections most commonly occurring during summer months, the time of year this tick stage is most active [71]. 

### 2.6. Rickettsia rickettsii

*Rickettsia rickettsii*, the causative agent of Rocky Mountain spotted fever (RMSF), is found in ticks from southern Canada into parts of northern South America and is the predominant tick-borne rickettsiosis in North America. There is limited epidemiologic understanding of RMSF in Canada because it has not been a reportable disease in the country since 1978 (http://diseases.canada.ca/notifiable/diseases-list). The disease has been nationally notifiable in the United States since 1920, with most cases reported from south Atlantic (North Carolina) and south-central states (Arkansas, Missouri, Oklahoma and Tennessee) (https://www.cdc.gov/otherspottedfever/stats/index.html). RMSF is a notifiable disease in Mexico as well, with epidemics in the past decade concentrated along the United States-Mexico border region [75]. 

Classic clinical manifestations of RMSF disease consist of fever, headache and a characteristic petechial rash which develops approximately a week into illness and often involves the palms and soles [12]. The case fatality rate in the 1930s was 24%, decreasing to 3% after the discovery and widespread use of antibiotics (1940s) and by 2007 was only 0.3% [76]. The virulence of a given *R. rickettsii* strain can be influenced by abiotic (ambient temperature) and biotic (geographic strain, host factors, feeding status of tick) factors [12], including possible blockage of transovarial infection and transmission interference by *Rickettsia peacockii* [8,77,78]. Human populations at highest risk for fatal infection include the young, elderly, immunosuppressed and Native American Indians [76]. Dogs are susceptible to infection with *R. rickettsii* and often exhibit symptoms of anorexia, lethargy and petechiae in the oral membranes [42]. While dogs are vertebrate reservoirs of *R. rickettsii*, the level of infectivity for the tick vector is low, indicating that dogs may be more involved in increasing the tick-human encounter rate than amplifying the infection [79].

Historically, the principal tick vectors of *R. rickettsii* were *Dermacentor* spp. with *D. variabilis* in eastern and central United States and *Dermacentor andersoni* in western states. A field investigation following a 2003–2004 outbreak in eastern Arizona, in which 11 confirmed (2 fatal) and five probable human infections were identified, implicated *R. sanguineus* as a vector of RMSF [80]. All ticks collected were *R. sanguineus*, with 3% of ticks collected in the environment and a single tick collected off a patient’s dog testing positive by PCR and culture. This tick species is implicated as a key vector in the desert southwestern United States, Mexico and South America [12]. *Amblyomma americanum* may also transmit *R. rickettsii* but its role as a vector in North America is likely minor [81]. In Mexico, *A. cajenennse*, *A. maculatum*, *A. americanum* and *Dermacentor nitens* have also tested positive for *R. rickettsii* [31]; however, the role of these other species in transmission of RMSF is unknown. 

## 3. Flea-Borne Rickettsiae (in Alphabetical Order)

### 3.1. Rickettsia felis

*Rickettsia felis*, the etiologic agent of flea-borne spotted fever, was first suspected to be a human pathogen in 1991 after its detection in the blood of a Texas patient diagnosed with murine typhus [82]. *Rickettsia felis* appears to share the same suburban transmission cycle as *Rickettsia typhi* with the cat flea, *Ctenocephalides felis*, serving as the primary vector and domestic animals and urban wildlife as potential reservoir hosts. *Ctenocephalides felis* in California and Oklahoma have been shown to be co-infected with both agents, though *R. felis* infection prevalence is generally higher [83,84]. Additionally, Noden et al. [85] demonstrated that *C. felis* naturally infected with *R. felis* could acquire and maintain *R. typhi* infections under experimental conditions. Dual infections were found in 13–50% of examined fleas. It is suspected that *C. felis* acquire the organism by feeding on a *R. felis* infected host, such as a cat or opossum but this step of the transmission cycle has yet to be confirmed. Cat fleas are able to maintain the infection through at least 12 generations without feeding on another rickettsemic host [86], suggesting that transmission among *C. felis* could be through co-feeding [87] or mating [9]. 

Since 1991, *R. felis* has been identified in the blood or skin biopsy of six patients residing in Yucatán, Mexico [88,89,90] but has not been reported in a patient in the United States since the initial finding. Diagnoses have been based only on molecular or serologic detection methods [91,92,93] and due to the lack of an isolate or culture from a clinical specimen, uncertainties have arisen regarding the pathogenicity of this organism. Vertical transmission of *R. felis* in a non-hematophagous insect species—book lice (*Liposcelis bostrychophila)*—as well as the presence of *R. felis* DNA in a wide variety of arthropods further suggests that this organism is non-pathogenic and likely serves as an endosymbiont similar to *Wolbachia* [91,92]. 

### 3.2. Rickettsia typhi

Outside of the contiguous United States, *R. typhi* is endemic in port cities and urban centers where large populations of commensal rodents are concentrated [94]. *Rickettsia typhi*, the causative agent of endemic typhus, is maintained in these locations in a rat-flea-rat cycle involving two rat species, *Rattus rattus* and *Rattus norvegicus* and the Oriental rat flea, *Xenopsylla cheopis*. Between 1916 and 1945, 394 human cases of typhus, including 21 deaths, were reported in southern California resulting in an intense public health intervention campaign in the 1920s to remove rodents in the city of Los Angeles [95]. Though sporadic cases did occur, a decline in the number of cases in the county was attributed to the removal of rats. In the 1950s, however, there was a shift in the distribution of the disease as cases were located in the foothill and suburban areas and not the highly urban centers [96]. Field investigations conducted in suburban neighborhoods around patient residences failed to recover serologically-positive rodents, suggesting the likely involvement of a different arthropod vector and reservoir host. In the same study, 8 (11%) of 75 opossums—*Didelphis virginianus*—were seroreactive to *R. typhi* antigens and the organism was isolated from the spleen of one of the seropositive opossums. Additionally, opossums were heavily infested with *C. felis*, though *R. typhi* was not detected in any ectoparasites [96]. Similar findings were reported in southern Texas in 1970 with anecdotal evidence that 10 (36%) of 28 murine typhus cases the previous year were likely acquired after contact with cats and/or *C. felis* [97]. One case-patient’s cat was seroreactive to *R. typhi* antigen leading to the proposed novel suburban transmission cycle involving cat fleas and cats/opossums. It is now known that murine typhus occurs in distinct urban and suburban cycles. 

The majority of human cases reported today in southern California and Texas in the United States, as well as parts of Mexico, are associated with contact with *C. felis* and their hosts, i.e., domestic animals and urban wildlife [92,98,99]. Between 2003 and 2013, there were over 2000 human cases reported in Texas and California [100], (https://www.cdph.ca.gov/Programs/CID/DCDC/CDPH%20Document%20Library/Flea-borneTyphusCaseCounts.pdf). Reasons for the geographic concentration of cases in these areas remain unclear as the vector and reservoir hosts are relatively ubiquitous. Murine typhus is not a nationally notifiable disease in the United States and one likely factor may be underdiagnoses/misdiagnoses in non-endemic regions [101]. A study conducted in Mexico City, Mexico, demonstrated a 15% (207 of 1382 individuals tested) seroprevalence of *R. typhi* in healthy adult blood donors, suggesting contact with *R. typhi* vectors and reservoir hosts may be relatively common in portions of North America [102]. Additionally, 7% (6/90) of Norway rats, *R. norvegicus*, trapped in an urban setting in Maryland were shown to harbor *R. typhi* antibodies, providing further evidence that the potential for infection is possibly more widespread than reported [103].

Like other flea-borne rickettsiae, humans become infected with *R. typhi* through the infectious bite of a flea or through scarification of infectious flea fecal material [104]. The prevalence of *R. typhi* in fleas in North America varies greatly: 0–2% of *C. felis* in California [82,105,106], 0–7% of *C. felis* in Texas [99,107,108] and 2% of *X. cheopis* in Hawaii [109]. Though *X. cheopis* and *C. felis* are the presumed vectors in urban and suburban areas, respectively, *R. typhi* DNA has been detected in a variety of other arthropods including other flea species, lice and ticks [83,110,111,112]. However, experimental studies are needed to elucidate the role these other vectors might play in the transmission of this disease.

## 4. Louse-Borne Rickettsia

### Rickettsia prowazekii

Outbreaks of *Rickettsia prowazekii*, the agent of epidemic typhus, are usually only observed in individuals living in crowded and unhygienic conditions [113,114]. *Rickettsia prowazekii* is transmitted to humans, the primary reservoir, by the body louse *Pediculus humanus humanus*. Transmission occurs by inoculation of infectious *P. humanus humanus* fecal material into the bite site, conjunctivae, or mucous membranes [113]. *Rickettsia prowazekii* is lethal to the louse, commonly killing the insect within two weeks after acquisition of an infected blood meal, due to damage of midgut epithelial cells [115]. Since *R. prowazekii* is not passaged transovarially, *P. humanus humanus* must feed on an infected host and infect a naïve host to continue the transmission cycle [114]. People infected with *R. prowazekii* often develop a very high fever; the increase in body temperature causes the louse to seek a new host, thus aiding in transmission of the pathogen [114]. In addition to high fever, most patients present with non-specific clinical symptoms including malaise and rash. Some individuals may develop more severe manifestations including seizures, confusion and coma [114]. If untreated, disease fatality ranges from 13% to 30% [114]. For individuals that do not receive treatment but survive, some exhibit post-infection reactivation, i.e., Brill-Zinsser disease, months or years later, typically with milder symptoms than the original infection. These individuals may also serve as sources for new outbreaks. 

Though rare, most cases of *R. prowazekii* reported in North America are not associated with lice but rather contact with the southern flying squirrel, *Glaucomys volans* [116,117,118]. The exact mode of transmission for this form of the disease, known more commonly as sylvatic epidemic typhus, is unknown, but one-third of patients report direct or indirect interaction with flying squirrels [118]. As flying squirrel ectoparasites may be highly host-specific, it is more likely transmission occurs through inhalation or mucosal contact with infectious arthropod feces present in the nesting material rather than through the bite of an infected arthropod [118]. Association with flying squirrels or lice could not be established for one reported case in an individual from New Mexico with no travel history to the eastern United States, the known range of the southern flying squirrel [119]. Although unconfirmed, it has been suggested that additional arthropods, such as *Amblyomma* spp. ticks, may serve as vectors of *R. prowazekii* [120]. 

## 5. Mite-Borne Rickettsiae

### Rickettsia akari

Rickettsialpox, the disease caused by infection with *Rickettsia akari*, is transmitted to humans by the bite of the house mouse mite, *Liponyssoides sanguineus* [121]. First described in New York City in 1946, 100 to 200 cases were reported each year in the United States during the 1940s and 1950s, with poor housing conditions promoting house mite infestations and thus rickettsialpox outbreaks in large metropolitan areas [121]. An eschar may form at the site of inoculation and febrile symptoms, along with fatigue, myalgia, sweats and chills, often develop within a week of an infectious bite [121]. In the 1950s, control measures were implemented for the house mouse (*Mus musculus*)—the primary host of the mite—and this resulted in a decline in cases. While *M. musculus* generally serves as the vertebrate reservoir, sylvatic and commensal rodents in Orange County, California and domestic dogs in New York City have been shown to be serologically positive for *R. akari* [122,123] suggesting *L. sanguineus* may feed on a broader host range than previously thought [124]. 

Between 1989 and 2000, a median of one confirmed case was reported annually in New York City [125]. Though outbreaks have been recorded recently in parts of the United States and Mexico [125,126], case counts likely do not reflect the true disease incidence. Sporadic cases may be misdiagnosed in areas where the disease is unfamiliar to physicians or is not endemic [127]. Additionally, individuals that have frequent contact with commensal rodents and their ectoparasites, such as the homeless and intravenous drug users (IDUs), may be less likely to receive medical care and thereby go undiagnosed. Studies conducted in New York City and Baltimore, Maryland found that IDUs were at increased risk for *R. akari* infection; 9% (18/204) and 16% (102/631) of users were seroreactive to *R. akari*, respectively [128,129]. A similar seroprevalence, 8% (25/299), was found in individuals that visited a free clinic on ‘Skid Row’ in downtown Los Angeles, California [123]. Those that are economically disadvantaged, the urban poor, are potentially at greatest risk for acquiring this disease [130]. 

## 6. Conclusions

Identification and accurate diagnosis of rickettsial infections can be challenging. Phylogenetic relationships between and within biotypes are unresolved and add to the complexity of determining transmission cycles, vertebrate hosts, deducing pathogenicity based on relatedness, and basic development of diagnostic testing protocols [131]. Cross-reactivity has been reported among spotted fever and typhus group rickettsiae and antibody tests demonstrate prior exposure and not active infection [132]. Confirmatory diagnosis requires an acute and convalescent serum sample, ideally 14 or more days apart, to confirm an illness is due to a rickettsiosis [4]. To accurately identify the *Rickettsia* species, cross-adsorption assays using two or more antigens are necessary but these types of tests are expensive and time-consuming [132]. Though molecular methods and culture are the preferred method for diagnosis, they also have drawbacks. As isolation of organisms from clinical specimens must be conducted in a biosafety level 3 laboratory (BSL-3) [132], the number of hospitals and laboratories able to perform such work is limited. In addition, collection of clinical specimens, such as whole blood, eschar biopsy, or eschar/skin swab, must be initiated prior to antibiotic treatment. As discussed by Labruna and Walker [91], the sensitivity of the PCR assay using whole blood depends upon the number of rickettsiae circulating in the bloodstream—the lower the magnitude of vasculitic lesions, the more likely false negatives will be reported. As such, highly pathogenic rickettsiae, such as *R. conorii* (Mediterranean spotted fever) and *R. rickettsii* (RMSF), often go undetected in blood samples unless in a fatal case. The presence of DNA in clinical samples and ectoparasites does not verify viability or infectivity of the organism. The culture of live organisms, however, is imperative for demonstration of pathogenicity and transmissibility, characterization of novel rickettsiae and development of diagnostic tools [132]. 

The number of rickettsiae detected in North America appears to be increasing yearly but whether these new or *Candidatus* species are pathogenic to humans remains unknown. *Candidatus* Rickettsia andeanae has been found in *A. maculatum* in Kansas, Mississippi, Oklahoma, Tennessee and Virginia in the United States [22,59,133,134,135] and in Ontario, Canada [136]. As Ca. R. andeanae has been isolated from *A. maculatum* embryonic cells [137], it is likely this organism is passaged transovarially and serves as an endosymbiont of the Gulf Coast tick. Paddock et al. [133] suggest that infection with Ca. R. andeanae excludes infection with *R. parkeri* similar to what occurs between *R. peacockii* and *R. rickettsii* infection in *D. andersoni* [138,139]. Unsuccessful attempts to culture *R. peacockii* have eluded the cause of transmission interference observed between *R. peacockii* and *R. rickettsii* [138], which could be due to *R. peacockii* outcompeting *R. rickettsii* within the tick tissues, or could be an effect of tick population dynamics whereby *R. rickettsii*-infected ticks exhibit increased mortality and reduced fecundity relative to *R. peacockii*-infected ticks [139]. Mounting evidence supports rickettsial competition and exclusion within the tick tissues (primarily ovaries) as the driver for transmission interference [139]. Preliminary research on *D. andersoni* from North America shows that simultaneous infection with the endosymbiont *Rickettsia bellii* is negatively correlated to *Anaplasma marginale* (etiological agent of anaplasmosis) acquisition [140]. Additionally, *Rickettsia rhipicephali* may serve as a protective endosymbiont to *Dermacentor* spp. by altering the molecular expression of oocytes to prevent infection with a secondary rickettsiae such as seen experimentally for *R. montanensis* [141], or by eliciting a cross-protective immune response such as seen for *R. rickettsii* [142]. *Rickettsia peacockii*, *R. bellii* and *R. rhipicephali* as well as other rickettsial endosymbionts have been reported in ticks from the United States [71,138,140,143,144]. Two flea-associated rickettsial species—*Candidatus* Rickettsia senegalensis and/or *Rickettsia asembonensis*—have also been reported in fleas and ticks in the United States and Costa Rica [84,99,105,145] but their role in transmission dynamics is still unknown. 

As vectors, such as ticks and fleas, expand into new geographic regions, the number of rickettsial infections and other arthropod-borne diseases reported annually from North America is likely to increase [28]. For example, the increased incidence of spotted fever group rickettsiosis cases in the United States appears associated with the expanding distribution of the Lone Star tick, *A. americanum* [25,28]. Importation of invasive arthropods [60,146] is also of great concern due to the potential introduction and establishment of novel zoonotic pathogens. Due to ecological, biological, and behavioral factors promoting contact between humans and disease-carrying arthropods, it is important to continue researching, documenting, and monitoring vector-borne rickettsioses throughout North America.

## Figures and Tables

**Table 1:** Suspected or confirmed vector-borne *Rickettsia* species reported in North America associated with human diseases, in alphabetical order.

Rickettsia Species	Disease	Confirmed and/or Suspected Primary Arthropod Vector(s) ^a^	Vector Host(s)	Distribution	Primary Clinical Manifestations
*R. akari*	Rickettsialpox	Mite: *Liponyssoides sanguineus*	House mouse, other rodents	Major urban centers	Fever, eschar, papulovesicular rash
*R. amblyommatis*	N/A ^b^	Tick: *Amblyomma americanum*	Birds, rodents, companion animals, wildlife	Southeastern USA	Fever, headache, myalgia, rash
*R. felis*	Flea-borne spotted fever	Flea: *Ctenocephalides felis*	Companion animals, urban wildlife	Southern California, Texas, Hawaii USA; Mexico	Fever, headache, rash
*R. massiliae* ^c^	Mediterranean spotted fever-like disease	Tick: *Rhipicephalus sanguineus*	Dogs	ND ^d^	Maculopapular rash including soles, headache, nausea
*R. montanensis*	N/A ^b^	Tick: *Dermacentor variabilis*	Rodents, companion animals, wildlife	ND—likely midwestern and mid-Atlantic USA states	Maculopapular rash including palms and soles, eschar
*R. parkeri*	Maculatum infection, Tidewater spotted fever, American boutonneuse fever	Tick: *Amblyomma maculatum*	Mammals, birds	Southern portions of USA	Fever, headache, malaise, myalgia/arthralgia, eschar, maculopapular rash
*R. philipii*	Pacific Coast tick fever	Tick: *Dermacentor occidentalis*	Rodents, companion animals, urban wildlife	California USA	Fever, headache, eschar, maculopapular rash including palms and soles
*R. prowazekii*	Epidemic typhus, sylvatic typhus	Lice: *Pediculus humanus humanus* Southern flying squirrel ectoparasites	Humans, flying squirrels	Southeastern USA	Fever, eschar, headache, lymphadenopathy
*R. rickettsii*	Rocky Mountain spotted fever	Ticks: *Amblyomma* species, *Dermacentor* species, *Rhipicephalus sanguineus*	Small mammals, companion animals	Southeastern and southwestern USA; Mexico	Fever, headache, myalgia, malaise, rash (typically not on palms and soles)
*R. typhi*	Murine typhus, endemic typhus	Fleas: *Xenopsylla cheopis*, *Ctenocephalides felis*	Rodents, companion animals, urban wildlife	Southern California, Texas, and Hawaii USA; Mexico	Fever, headache, maculopapular rash including palms and soles

^a^ Based on the detection of rickettsiae DNA in an arthropod or confirmed laboratory experimental transmission studies; ^b^ No confirmed human cases. Previous reports based solely on serological findings; ^c^ No human cases reported, however, DNA has been detected in *R. sanguineus*; ^d^ Not determined/Unknown.

## References

[1] Faccini-Martínez Á.A., García-Álvarez L., Hidalgo M., Oteo J.A. (2014). Syndromic classification of rickettsioses: An approach for clinical practice. Int. J. Infect. Dis..

[2] Raoult D. (2010). Emerging rickettsioses reach the United States. Clin. Infect. Dis..

[3] Eremeeva M.E., Dasch G.A. (2015). Challenges posed by tick-borne rickettsiae: Eco-epidemiology and public health implications. Front. Public Health.

[4] Richards A.L. (2012). Worldwide detection and identification of new and old rickettsiae and rickettsial diseases. FEMS. Immunol. Med. Microbiol..

[5] Mahajan S.K. (2012). Rickettsial diseases. J. Assoc. Phys. India.

[6] Burgdorfer W. (1970). Hemolymph test: A technique for detection of rickettsiae in ticks. Am. J. Trop. Med. Hyg..

[7] Adams J.R., Schmidtmann E.T., Azad A.F. (1990). Infection of colonized cat fleas, *Ctenocephalides felis* (Bouché), with a rickettsia-like microorganism. Am. J. Trop. Med. Hyg..

[8] Azad A.F., Beard C.B. (1998). Rickettsial pathogens and their arthropod vectors. Emerg. Infect. Dis..

[9] Hirunkanokpun S., Thepparit C., Foil L.D., Macaluso K.R. (2011). Horizontal transmission of *Rickettsia felis* between cat fleas, *Ctenocephalides felis*. Mol. Ecol..

[10] Brown L.D., Christofferson R.C., Banajee K.H., Del Piero F., Foil L.D., Macaluso K.R. (2015). Cofeeding intra- and interspecific transmission of an emerging insect-borne rickettsial pathogen. Mol. Ecol..

[11] Biggs H.M., Behravesh C.B., Bradley K.K., Dahlgren F.S., Drexler N.A., Dumler J.S., Folk S.M., Kato C.Y., Lash R.R., Levin M.L. (2016). Diagnosis and management of tickborne rickettsial diseases: Rocky Mountain spotted fever and other spotted fever group rickettsioses, ehrlichioses, and anaplasmosis—United States. MMWR Recomm. Rep..

[12] Chen L.F., Sexton D.J. (2008). What’s new in Rocky Mountain spotted fever?. Infect. Dis. Clin. N. Am..

[13] Karpathy S.E., Slater K.S., Goldsmith C.S., Nicholson W.L., Paddock C.D. (2016). *Rickettsia amblyommatis* sp. nov., a spotted fever group *Rickettsia* associated with multiple species of *Amblyomma* ticks in North, Central and South America. Int. J. Syst. Evol. Microbiol..

[14] Gleim E.R., Garrison L.E., Vello M.S., Savage M.Y., Lopez G., Berghaus R.D., Yabsley M.J. (2016). Factors associated with tick bites and pathogen prevalence in ticks parasitizing humans in Georgia, USA. Parasit. Vectors.

[15] Billeter S.A., Blanton H.L., Little S.E., Levy M.G., Breitschwerdt E.B. (2007). Detection of “*Rickettsia amblyommii*” in association with a tick bite rash. Vector Borne Zoonotic Dis..

[16] Nicholson W.L., Masters E., Wormser G.P. (2009). Preliminary serologic investigation of “*Rickettsia amblyommii*” in the aetiology of southern tick associated rash illness (STARI). Clin. Microbiol. Infect..

[17] Vaughn M.F., Delisle J., Johnson J., Daves G., Williams C., Reber J., Mendell N.L., Bouyer D.H., Nicholson W.L., Moncayo A.C. (2014). Seroepidemiologic study of human infection with spotted fever group rickettsiae in North Carolina. J. Clin. Microbiol..

[18] Delisle J., Mendell N.L., Stull-Lane A., Bloch K.C., Bouyer D.H., Moncayo A.C. (2016). Human infections by multiple spotted fever group rickettsiae in Tennessee. Am. J. Trop. Med. Hyg..

[19] Apperson C.S., Engber B., Nicholson W.L., Mead D.G., Engel J., Yabsley M.J., Dail K., Johnson J., Watson D.W. (2008). Tick-borne diseases in North Carolina: Is “*Rickettsia amblyommii*” a possible cause of rickettsiosis reported as Rocky Mountain spotted fever?. Vector Borne Zoonotic Dis..

[20] Stomdahl E.Y., Vince M.A., Billingsley P.M., Dobbs N.A., Williamson P.C. (2008). *Rickettsia amblyommii* infecting *Amblyomma americanum* larvae. Vector Borne Zoonotic Dis..

[21] Hermance M., Marques dos Santos R.I., Heinze D., Hausser N., Bouyer D.H., Thangamani S. (2014). Detection of *Rickettsia amblyommii* in ticks collected from Missouri, USA. Emerg. Microbes Infect..

[22] Nadolny R.M., Wright C.L., Sonenshine D.E., Hynes W.L., Gaff H.D. (2014). Ticks and spotted fever group rickettsiae of southeastern Virginia. Ticks Tick Borne Dis..

[23] Hudman D.A., Sargentini N.J. (2016). Detection of *Borrelia*, *Ehrlichia*, and *Rickettsia* spp. in ticks in northeast Missouri. Ticks Tick Borne Dis..

[24] Miller M.K., Jiang J., Truong M., Yarina T., Evans H., Christensen T.P., Richards A.L. (2016). Emerging tick-borne Rickettsia and Ehrlichia at Joint Base Langley-Eustis, Fort Eustis, Virginia. US Army Med. Dep. J..

[25] Monzón J.D., Atkinson E.G., Henn B.M., Benach J.L. (2016). Population and evolutionary genomics of *Amblyomma americanum*, an expanding arthropod disease vector. Genome Biol. Evol..

[26] Jiang J., Yarina T., Miller M.K., Stromdahl E.Y., Richards A.L. (2010). Molecular detection of *Rickettsia amblyommii* in *Amblyomma americanum* parasitizing humans. Vector Borne Zoonotic Dis..

[27] Wallace J.W., Nicholson W.L., Perniciaro J.L., Vaughn M.F., Funkhouser S., Juliano J.J., Lee S., Kakumanu M.L., Ponnusamy L., Apperson C.S. (2016). Incident tick-borne infections in a cohort of North Carolina outdoor workers. Vector Borne Zoonotic Dis..

[28] Dahlgren F.S., Paddock C.D., Springer Y.P., Eisen R.J., Behravesh C.B. (2016). Expanding range of *Amblyomma americanum* and simultaneous changes in the epidemiology of spotted fever group rickettsiosis in the United States. Am. J. Trop. Med. Hyg..

[29] Hun L., Troyo A., Taylor L., Barbieri A.M., Labruna M.B. (2011). First report of the isolation and molecular characterization of *Rickettsia amblyommii* and *Rickettsia felis* in Central America. Vector Borne Zoonotic Dis..

[30] Sánchez-Montes S., Ríos-Muñoz C.A., Espinosa-Martínez D.V., Guzmán-Cornejo C., Berzunza-Cruz M., Becker I. (2016). First report of “*Candidatus* Rickettsia amblyommii” in west coast of Mexico. Ticks Tick Borne Dis..

[31] Sosa-Gutierrez C.G., Vargas-Sandoval M., Torres J., Gordillo-Pérez G. (2016). Tick-borne rickettsial pathogens in questing ticks, removed from humans and animals in Mexico. J. Vet. Sci..

[32] Vitale G., Mansuelo S., Rolain J.-M., Raoult D. (2006). *Rickettsia massiliae* human isolation. Emerg. Infect. Dis..

[33] García-García J.C., Portillo A., Núñez M.J., Santibáñez S., Castro B., Oteo J.A. (2010). Case Report: A patient from Argentina infected with *Rickettsia massiliae*. Am. J. Trop. Med. Hyg..

[34] Beati L., Finidori J.-P., Gilot B., Raoult D. (1992). Comparison of serologic typing, sodium dodecyl sulfate-polyacrylaminde gel electrophoresis protein analysis, and genetic restriction fragment length polymorphism analysis for identification of rickettsiae: Characterization of two new rickettsial strains. J. Clin. Microbiol..

[35] Dupont H.T., Cornet J.-P., Raoult D. (1994). Identification of rickettsiae from ticks collected in the Central African Republic using the polymerase chain reaction. Am. J. Trop. Med. Hyg..

[36] Eremeeva M.E., Bosserman E.A., Demma L.J., Zambrano M.L., Blau D.M., Dasch G.A. (2006). Isolation and identification of *Rickettsia massiliae* from *Rhipicephalus sanguineus* ticks collected in Arizona. Appl. Environ. Microbiol..

[37] Beeler E., Abramowicz K.F., Zambrano M.L., Sturgeon M.M., Khalaf N., Hu R., Dasch G.A., Eremeeva M.E. (2011). A focus of dogs and *Rickettsia massiliae*-infected *Rhipicephalus sanguineus* in California. Am. J. Trop. Med. Hyg..

[38] Fornadel C.M., Smith J.D., Zawada S.E., Arias J.R., Norris D.E. (2013). Detection of *Rickettsia massiliae* in *Rhipicephalus sanguineus* from the eastern United States. Vector Borne Zoonotic Dis..

[39] Parola P., Socolovschi C., Jeanjean L., Bitam I., Fournier P.-E., Sotto A., Labauge P., Raoult D. (2008). Warmer weather linked to tick attack and emergence of severe rickettsioses. PLoS Negl. Trop. Dis..

[40] McQuiston J.H., Zemtsova G., Perniciaro J., Hutson M., Singleton J., Nicholson W.L., Levin M.L. (2012). Afebrile spotted fever group *Rickettsia* infection after a bite from a *Dermacentor variabilis* tick infected with *Rickettsia montanensis*. Vector Borne Zoonotic Dis..

[41] Centers for Disease Control and Prevention Statistics and Epidemiology Rocky Mountain Spotted Fever (RMSF). https://www.cdc.gov.

[42] Breitschwerdt E.B., Walker D.H., Levy M.G., Burgdorfer W., Corbett W.T., Hurlbert S.A., Stebbins M.E., Curtis B.C., Allen D.A. (1988). Clinical, hematologic, and humoral immune response in female dogs inoculated with *Rickettsia rickettsii* and *Rickettsia montana*. Am. J. Vet. Res..

[43] Barrett A., Little S.E., Shaw E. (2014). “*Rickettsia amblyommii*” and *R. montanensis* infection in dogs following natural exposure to ticks. Vector Borne Zoonotic Dis..

[44] Pagac B.B., Miller M.K., Mazzei M.C., Nielsen D.H., Jiang J., Richards A.L. (2014). *Rickettsia parkeri* and *Rickettsia montanensis*, Kentucky and Tennessee, USA. Emerg. Infect. Dis..

[45] Ammerman N.C., Swanson K.I., Anderson J.M., Schwartz T.R., Seaberg E.C., Glass G.E., Norris D.E. (2004). Spotted-fever group *Rickettsia* in *Dermacentor variabilis*, Maryland. Emerg. Infect. Dis..

[46] Dergousoff S.J., Gajadhar A.J., Chilton N.B. (2009). Prevalence of *Rickettsia* species in Canadian populations of *Dermacentor andersoni* and *D. variabilis*. Appl. Environ. Microbiol..

[47] Yunik M.E., Galloway T.D., Lindsay L.R. (2015). Assessment of prevalence and distribution of spotted fever group rickettsiae in Manitoba, Canada, in the American dog tick, *Dermacentor variabilis* (Acari: Ixodidae). Vector Borne Zoonotic Dis..

[48] St John H.K., Adams M.L., Masuoka P.M., Flyer-Adams J.G., Jiang J., Rozmajzl P.J., Stromdahl E.Y., Richards A.L. (2016). Prevalence, distribution, and development of an ecological niche model of *Dermacentor variabilis* ticks positive for *Rickettsia montanensis*. Vector Borne Zoonotic Dis..

[49] Parker R.R., Kohls G.M., Cox G.W., Davis G.E. (1939). Observations on the infectious agent from *Amblyomma maculatum*. Public Health Rep..

[50] Paddock C.D., Sumner J.W., Comer J.A., Zaki S.R., Goldsmith C.S., Goddard J., McLellan S.L., Tamminga C.L., Ohl C.A. (2004). *Rickettsia parkeri*: A newly recognized cause of spotted fever rickettsiosis in the United States. Clin. Infect. Dis..

[51] Whitman T.J., Richards A.L., Paddock C.D., Tamminga C.L., Sniezek P.J., Jiang J., Byers D.K., Sanders J.W. (2007). *Rickettsia parkeri* infection after tick bite, Virginia. Emerg. Infect. Dis..

[52] Paddock C.D., Finley R.W., Wright C.S., Robinson H.N., Schrodt B.J., Lane C.C., Ekenna O., Blass M.A., Tamminga C.L., Ohl C.A. (2008). *Rickettsia parkeri* rickettsiosis and its clinical distinction from Rocky Mountain spotted fever. Clin. Infect. Dis..

[53] Cragun W.C., Bartlett B.L., Ellis M.W., Hoover A.Z., Tyring S.K., Mendoza N., Vento T.J., Nicholson W.L., Eremeeva M.E., Olano J.P. (2010). The expanding spectrum of eschar-associated rickettsioses in the United States. Arch. Dermatol..

[54] Herrick K.L., Pena S.A., Yaglom H.D., Layton B.J., Moors A., Loftis A.D., Condit M.E., Singleton J., Kato C.Y., Denison A.M. (2016). *Rickettsia parkeri* rickettsiosis, Arizona, USA. Emerg. Infect. Dis..

[55] Straily A., Feldpausch A., Ulbrich C., Schell K., Casillas S., Zaki S.R., Denison A.M., Condit M., Gabel J., Paddock C.D. (2016). Notes from the field: *Rickettsia parkeri* rickettsiosis—Georgia, 2012–2014. MMWR Morb. Mortal. Wkly. Rep..

[56] Sumner J.W., Durden L.A., Goddard J., Stromdahl E.Y., Clark K.L., Reeves W.K., Paddock C.D. (2007). Gulf Coast ticks (*Amblyomma maculatum*) and *Rickettsia parkeri*, United States. Emerg. Infect. Dis..

[57] Paddock C.D., Fournier P.-E., Sumner J.W., Goddard J., Elshenawy Y., Metcalfe M.G., Loftis A.D., Varela-Stokes A. (2010). Isolation of *Rickettsia parkeri* and identification of a novel spotted fever group *Rickettsia* sp. from Gulf Coast ticks (*Amblyomma maculatum*) in the United States. Appl. Environ. Microbiol..

[58] Varela-Stokes A.S., Paddock C.D., Engber B., Toliver M. (2011). *Rickettsia parkeri* in *Amblyomma maculatum* ticks, North Carolina, USA, 2009–2010. Emerg. Infect. Dis..

[59] Ferrari F.A., Goddard J., Paddock C.D., Varela-Stokes A.S. (2012). *Rickettsia parkeri* and *Candidatus* Rickettsia andeanae in Golf Coast ticks, Mississippi, USA. Emerg. Infect. Dis..

[60] Mertins J.W., Moorhouse A.S., Alfred J.T., Hutcheson H.J. (2010). *Amblyomma triste* (Acari: Ixodidae): New North American collection records, including the first from the United States. J. Med. Entomol..

[61] Grasperge B.J., Wolfson W., Macaluso K.R. (2012). *Rickettsia parkeri* infection in domestic dogs, southern Louisiana, USA, 2011. Emerg. Infect. Dis..

[62] Moraru G.M., Goddard J., Murphy A., Link D., Belant J.L., Varela-Stokes A. (2013). Evidence of antibodies to spotted fever group rickettsiae in small mammals and quail from Mississippi. Vector Borne Zoonotic Dis..

[63] Moraru G.M., Goddard J., Paddock C.D., Varela-Stokes A. (2013). Experimental infection of cotton rats and bobwhite quail with *Rickettsia parkeri*. Parasit. Vectors.

[64] Goddard J. (2003). Experimental infection of Lone Star ticks, *Amblyomma americanum* (L.), with *Rickettsia parkeri* and exposure of guinea pigs to the agent. J. Med. Entomol..

[65] Wright C.L., Sonenshine D.E., Gaff H.D., Hynes W.L. (2015). *Rickettsia parkeri* transmission to *Amblyomma americanum* by cofeeding with *Amblyomma maculatum* (Acari: Ixodidae) and potential for spillover. J. Med. Entomol..

[66] Henning T.C., Orr J.M., Smith J.D., Arias J.R., Norris D.E. (2014). Spotted fever group rickettsiae in multiple hard tick species from Fairfax County, Virginia. Vector Borne Zoonotic Dis..

[67] Philip R.N., Casper E.A., Burgdorfer W., Gerloff R.K., Hughes L.E., Bell E.J. (1978). Serologic typing of rickettsiae of the spotted fever group by microimmunofluorescence. J. Immunol..

[68] Philip R.N., Lane R.S., Casper E.A. (1981). Serotypes of tick-borne spotted fever group rickettsiae from western California. Am. J. Trop. Med. Hyg..

[69] Shapiro M.R., Fritz C.L., Tait K., Paddock C.D., Nicholson W.L., Abramowicz K.F., Karpathy S.E., Dasch G.A., Sumner J.W., Adem P.V. (2010). *Rickettsia* 364D: A newly recognized cause of eschar-associated illness in California. Clin. Infect. Dis..

[70] Johnston S.H., Glaser C.A., Padgett K., Wadford D.A., Espinosa A., Espinosa N., Eremeeva M.E., Tait K., Hobson B., Shtivelman S. (2013). *Rickettsia* spp. 364D causing a cluster of eschar-associated illness, California. Pediatr. Infect. Dis. J..

[71] Padgett K.A., Bonilla D., Eremeeva M.E., Glaser C., Lane R.S., Porse C.C., Castro M.B., Messenger S., Espinosa A., Hacker J. (2016). The eco-epidemiology of Pacific Coast tick fever in California. PLoS Negl. Trop. Dis..

[72] Furman D.P., Loomis E.C. (1984). The Ticks of California (Acari: Ixodidae).

[73] Wikswo M.E., Hu R., Dasch G.A., Krueger L., Arugay A., Jones K., Hess B., Bennett S., Kramer V., Eremeeva M.E. (2008). Detection and identification of spotted fever group rickettsiae in *Dermacentor* species from southern California. J. Med. Entomol..

[74] Billeter S.A., Yoshimizu M.H., Hu R. (2017). Species composition and temporal distribution of adult ixodid ticks and prevalence of *Borrelia burgdorferi* sensu lato and *Rickettsia* species in Orange County, California. J. Vector Ecol..

[75] Drexler N.A., Yaglom H., Casal M., Fierro M., Kriner P., Murphy B., Kjemtrup A., Paddock C.D. (2017). Fatal Rocky Mountain spotted fever along the United States-Mexico border, 2013–2016. Emerg. Infect. Dis..

[76] Dahlgren F.S., Holman R.C., Paddock C.D., Callinan L.S., McQuiston J.H. (2012). Fatal Rocky Mountain spotted fever in the United States, 1999–2007. Am. J. Trop. Med. Hyg..

[77] Burgdorfer W., Brinton L.P. (1975). Mechanisms of transovarial infection of spotted fever rickettsiae in ticks. Ann. N. Y. Acad. Sci..

[78] Felsheim R.F., Kurtti T.J., Munderloh U.G. (2009). Genome sequence of the endosymbiont *Rickettsia peacockii* and comparison with virulent *Rickettsia rickettsii*: Identificiation of virulence factors. PLoS ONE.

[79] Norment B.R., Burgdorfer W. (1984). Susceptibility and reservoir potential of the dog to spotted fever-group rickettsiae. Am. J. Vet. Res..

[80] Demma L.J., Traeger M.S., Nicholson W.L., Paddock C.D., Blau D.M., Eremeeva M.E., Dasch G.A., Levin M.L., Singleton J., Zaki S.R. (2005). Rocky Mountain spotted fever from an unexpected tick vector in Arizona. N. Engl. J. Med..

[81] Breitschwerdt E.B., Hegarty B.C., Maggi R.G., Lantos P.M., Aslett D.M., Bradley J.M. (2011). *Rickettsia rickettsii* transmission by a Lone Star tick, North Carolina. Emerg. Infect. Dis..

[82] Schriefer M.E., Sacci J.B., Dumler J.S., Bullen M.G., Azad A.F. (1994). Identification of a novel rickettsial infection in a patient diagnosed with murine typhus. J. Clin. Microbiol..

[83] Eremeeva M.E., Karpathy S.E., Krueger L., Hayes E.K., Williams A.M., Zaldivar Y., Bennett S., Cummings R., Tilzer A., Velten R.K. (2012). Two pathogens and one disease: Detection and identification of flea-borne rickettsiae in areas endemic for murine typhus in California. J. Med. Entomol..

[84] Noden B.H., Davidson S., Smith J.L., Williams F. (2017). First detection of *Rickettsia typhi* and *Rickettsia felis* in fleas collected from client-owned companion animals in the Southern Great Plains. J. Med. Entomol..

[85] Noden B.H., Radulovic S., Higgins J.A., Azad A.F. (1998). Molecular identification of *Rickettsia typhi* and *R. felis* in co-infected *Ctenocephalides felis* (Siphonaptera: Pulicidae). J. Med. Entomol..

[86] Wedincamp J., Foil L.D. (2002). Vertical transmission of *Rickettsia felis* in the cat flea (*Ctenocephalides felis* Bouché). J. Vector Ecol..

[87] Brown L.D., Banajee K.H., Foil L.D., Macaluso K.R. (2016). Transmission mechanisms of an emerging insect-borne rickettsial pathogen. Parasit. Vectors.

[88] Zavala-Velázquez J.E., Ruiz-Sosa J.A., Sánchez-Elias R.A., Becerra-Carmona G., Walker D.H. (2000). *Rickettsia felis* rickettsiosis in Yucatán. Lancet.

[89] Zavala-Velazquez J., Laviada-Molina H., Zavala-Castro J., Perez-Osorio C., Becerra-Carmona G., Ruiz-Sosa J.A., Bouyer D.H., Walker D.H. (2006). *Rickettsia felis*, the agent of an emerging infectious disease: Report of a new case in Mexico. Arch. Med. Res..

[90] Zavala-Castro J., Zavala-Velázquez J., Walker D., Pérez-Osorio J., Peniche-Lara G. (2009). Severe human infection with *Rickettsia felis* associated with hepatitis in Yucatan, Mexico. Int. J. Med. Microbiol..

[91] Labruna M.B., Walker D.H. (2014). *Rickettsia felis* and changing paradigms about pathogenic rickettsiae. Emerg. Infect. Dis..

[92] Billeter S.A., Metzger M.E. (2017). Limited evidence for *Rickettsia felis* as a cause of zoonotic flea-borne rickettsiosis in southern California. J. Med. Entomol..

[93] Blanton L.S., Walker D.H. (2017). Flea-borne rickettsioses and rickettsiae. Am. J. Trop. Med. Hyg..

[94] Civen R., Ngo V. (2008). Murine typhus: An unrecognized suburban vectorborne disease. Clin. Infect. Dis..

[95] Beck M.D., Van Allen A. (1947). Typhus fever in California, 1916–1945, inclusive; an epidemiologic and field laboratory study. Am. J. Hyg..

[96] Adams W.H., Emmons R.W., Brooks J.E. (1970). The changing ecology of murine (endemic) typhus in Southern California. Am. J. Trop. Med. Hyg..

[97] Older J.J. (1970). The epidemiology of murine typhus in Texas, 1969. J. Am. Med. Assoc..

[98] Dzul-Rosado K., González-Martínez P., Peniche-Lara G., Zavala-Velázquez J., Zavala-Castro J. (2013). Murine typhus in humans, Yucatan, Mexico. Emerg. Infect. Dis..

[99] Blanton L.S., Idowu B.M., Tatsch T.N., Henderson J.M., Bouyer D.H., Walker D.H. (2016). Opossums and cat fleas: New insights in the ecology of murine typhus in Galveston, Texas. Am. J. Trop. Med. Hyg..

[100] Murray K.O., Evert N., Mayes B., Fonken E., Erickson T., Garcia M.N., Sidwa T. (2017). Typhus group rickettsiosis, Texas, USA, 2003–2013. Emerg. Infect. Dis..

[101] Afzal Z., Kallumadanda S., Wang F., Hemmige V., Musher D. (2017). Acute febrile illness and complications due to murine typhus, Texas, USA. Emerg. Infect. Dis..

[102] Acuna-Soto R., Calderón-Romero L., Romero-López D., Bravo-Lindoro A. (2000). Murine typhus in Mexico City. Trans. R. Soc. Trop. Med. Hyg..

[103] Reeves W.K., Easterbrook J.D., Loftis A.D., Glass G.E. (2006). Serologic evidence for *Rickettsia typhi* and an ehrlichial agent in Norway rats from Baltimore, Maryland, USA. Vector Borne Zoonotic Dis..

[104] Azad A.F., Radulovic S., Higgins J.A., Noden B.H., Troyer J.M. (1997). Flea-borne rickettsioses: Ecological considerations. Emerg. Infect. Dis..

[105] Billeter S.A., Diniz P.P., Jett L.A., Wournell A.L., Kjemtrup A.M., Padgett K.A., Yoshimizu M.H., Metzger M.E., Barr M.C. (2016). Detection of *Rickettsia* species in fleas collected from cats in regions endemic and nonendemic for flea-borne rickettsioses in California. Vector Borne Zoonotic Dis..

[106] Maina A.N., Fogarty C., Krueger L., Macaluso K.R., Odhiambo A., Nguyen K., Farris C.M., Luce-Fedrow A., Bennett S., Jiang J. (2016). Rickettsial infections among *Ctenocephalides felis* and host animals during a flea-borne rickettsioses outbreak in Orange County, California. PLoS ONE.

[107] Schriefer M.E., Sacci J.B., Taylor J.P., Higgins J.A., Azad A.F. (1994). Murine typhus: Updated roles of multiple urban components and a second typhuslike rickettsia. J. Med. Entomol..

[108] Adjemian J., Parks S., McElroy K., Campbell J., Eremeeva M.E., Nicholson W.L., McQuiston J., Taylor J. (2010). Murine typhus in Austin, Texas, USA, 2008. Emerg. Infect. Dis..

[109] Eremeeva M.E., Warashina W.R., Sturgeon M.M., Buchholz A.E., Olmsted G.K., Park S.Y., Effler P.V., Karpathy S.E. (2008). *Rickettsia typhi* and *R. felis* in rat fleas (*Xenopsylla cheopis*), Oahu, Hawaii. Emerg. Infect. Dis..

[110] Reeves W.K., Nelder M.P., Korecki J.A. (2005). *Bartonella* and *Rickettsia* in fleas and lice from mammals in South Carolina, U.S.A. J. Vector Ecol..

[111] Peniche-Lara G., Dzul-Rosado K., Pérez-Osorio C., Zavala-Castro J. (2015). *Rickettsia typhi* in rodents and *R. felis* in fleas in Yucatán as a possible causal agent of undefined febrile cases. Rev. Inst. Med. Trop. Sao Paulo.

[112] Dzul-Rosado K., Lugo-Caballero C., Tello-Martin R., López-Avila K., Zavala-Castro J. (2017). Direct evidence of *Rickettsia typhi* infection in *Rhipicephalus sanguineus* ticks and their canine hosts. Open Vet. J..

[113] Badiaga S., Brouqi P. (2012). Human louse-transmitted infectious diseases. Clin. Microbiol. Infect..

[114] Angelakis E., Bechah Y., Raoult D. (2016). The history of epidemic typhus. Microbiol. Spectr..

[115] Gillepsie J.J., Ammerman N.C., Beier-Sexton M., Sobral B.S., Azad A.F. (2009). Louse- and flea-borne rickettsioses: Biological and genomic analyses. Vet. Res..

[116] Chapman A.S., Swerdlow D.L., Dato V.M., Anderson A.D., Moodie C.E., Marriott C., Amman B., Hennessey M., Fox P., Green D.B. (2009). Cluster of sylvatic epidemic typhus cases associated with flying squirrels, 2004–2006. Emerg. Infect. Dis..

[117] McQuiston J.H., Knights E.B., Demartino P.J., Paparello S.F., Nicholson W.L., Singleton J., Brown C.M., Massung R.F., Urbanowski J.C. (2010). Brill-Zinsser disease in a patient following infection with sylvatic epidemic typhus associated with flying squirrels. Clin. Infect. Dis..

[118] Prusinski M.A., White J.L., Wong S.J., Conlon M.A., Egan C., Kelly-Cirino C.D., Laniewicz B.R., Backenson P.B., Nicholson W.L., Eremeeva M.E. (2014). Sylvatic typhus associated with flying squirrels (*Glaucomys volans*) in New York State, United States. Vector Borne Zoonotic Dis..

[119] Massung R.F., Davis L.E., Slater K., McKechnie D.B., Puerzer M. (2001). Epidemic typhus meningitis in the southwestern United States. Clin. Infect. Dis..

[120] Medina-Sanchez A., Bouyer D.H., Alcantara-Rodriguez V., Mafra C., Zavala-Castro J., Whitworth T., Popov V.L., Fernandez-Salas I., Walker D.H. (2005). Detection of a typhus group *Rickettsia* in *Amblyomma* ticks in the state of Nuevo Leon, Mexico. Ann. N. Y. Acad. Sci..

[121] Boyd A.S. (1997). Rickettsialpox. Dermatol. Clin..

[122] Comer J.A., Vargas M.C., Poshni I., Childs J.E. (2001). Serologic evidence of *Rickettsia akari* infection among dogs in a metropolitan city. J. Am. Vet. Med. Assoc..

[123] Bennett S.G., Comer J.A., Smith H.M., Webb J.P. (2007). Serologic evidence of *Rickettsia akari*-like infection among wild-caught rodents in Orange County and humans in Los Angeles, California. J. Vector Ecol..

[124] Comer J.A., Paddock C.D., Childs J.E. (2001). Urban zoonoses caused by *Bartonella*, *Coxiella*, *Ehrlichia*, and *Rickettsia* species. Vector Borne Zoonotic Dis..

[125] Paddock C.D., Zaki S.R., Koss T., Singleton J., Sumner J.W., Comer J.A., Eremeeva M.E., Dasch G.A., Cherry B., Childs J.E. (2003). Rickettsialpox in New York City: A persistent urban zoonosis. Ann. N. Y. Acad. Sci..

[126] Zavala-Castro J.E., Zavala-Velázquez J.E., Peniche-Lara G.F., Sulú Uicab J.E. (2009). Human rickettsialpox, southeastern Mexico. Emerg. Infect. Dis..

[127] Krusell A., Comer J.A., Sexton D.J. (2002). Rickettsialpox in North Carolina: A case report. Emerg. Infect. Dis..

[128] Comer J.A., Diaz T., Vlahov D., Monterroso E., Childs J.E. (2001). Evidence of rodent-associated *Bartonella* and *Rickettsia* infections among intravenous drug users from Central and East Harlem, New York City. Am. J. Trop. Med. Hyg..

[129] Comer J.A., Tzianabos T., Flynn C., Vlahov D., Childs J.E. (1999). Serologic evidence of rickettsialpox (*Rickettsia akari*) infection among intravenous drug users in inner-city Baltimore, Maryland. Am. J. Trop. Med. Hyg..

[130] Leibler J.H., Zahour C.M., Gadhoke P., Gaeta J.M. (2016). Zoonotic and vector-borne infections among urban homeless and marginalized people in the United States and Europe, 1990–2014. Vector Borne Zoonotic Dis..

[131] Stothard D.R., Clark J.B., Fuerst P.A. (1994). Ancestral divergence of *Rickettsia bellii* from the spotted fever and typhus groups of *Rickettsia* and antiquity of the genus *Rickettsia*. Int. J. Syst. Bacteriol..

[132] Portillo A., de Sousa R., Santibáñez S., Duarte A., Edouard S., Fonseca I.P., Marques C., Novakova M., Palomar A.M., Santos M. (2017). Guidelines for the detection of *Rickettsia* spp.. Vector Borne Zoonotic Dis..

[133] Paddock C.D., Denison A.M., Dryden M.W., Noden B.H., Lash R.R., Abdelghani S.S., Evans A.E., Kelly A.R., Hecht J.A., Karpathy S.E. (2015). High prevalence of “*Candidatus* Rickettsia andeanae” and apparent exclusion of *Rickettsia parkeri* in adult *Amblyomma maculatum* (Acari: Ixodidae) from Kansas and Oklahoma. Ticks Tick Borne Dis..

[134] Mays S.E., Houston A.E., Trout Fryxell R.T. (2016). Specifying pathogen associations of *Amblyomma maculatum* (Acari: Ixodidae) in western Tennessee. J. Med. Entomol..

[135] Lee J.K., Moraru G.M., Stokes J.V., Wills R.W., Mitchell E., Unz E., Moore-Henderson B., Harper A.B., Varela-Stokes A.S. (2017). *Rickettsia parkeri* and “*Candidatus* Rickettsia andeanae” in questing *Amblyomma maculatum* (Acai: Ixodidae) from Mississippi. J. Med. Entomol..

[136] Wood H., Dillon L., Patel S.N., Ralevski F. (2016). Prevalence of *Rickettsia* species in *Dermacentor variabilis* ticks from Ontario, Canada. Ticks Tick Borne Dis..

[137] Ferrari F.A., Goddard J., Moraru G.M., Smith W.E., Varela-Stokes A.S. (2013). Isolation of “*Candidatus* Rickettsia andeanae” (Rickettsiales: Rickettsiaceae) in embryonic cells of naturally infected *Amblyomma maculatum* (Ixodida: Ixodidae). J. Med. Entomol..

[138] Niebylski M.L., Schrumpf M.E., Burgdorfer W., Fischer E.R., Gage K.L., Schwan T.G. (1997). *Rickettsia peacockii* sp. nov., a new species infecting wood ticks, *Dermacentor andersoni*, in western Montana. Int. J. Syst. Bacteriol..

[139] Niebylski M.L., Peacock M.G., Schwan T.G. (1999). Lethal effects of *Rickettsia rickettsii* on its tick vector (*Dermacentor andersoni*). Appl. Environ. Microbiol..

[140] Gall C.A., Reif K.E., Scoles G.A., Mason K.L., Mousel M., Noh S.M., Brayton K.A. (2016). The bacterial microbiome of *Dermacentor andersoni* ticks influences pathogen susceptibility. ISME J..

[141] Macaluso K.R., Sonenshine D.E., Ceraul S.M., Azad A.F. (2002). *Rickettsia* infection in *Dermacentor variabilis* (Acari: Ixodidae) inhibits transovarial transmission of a second *Rickettsia*. J. Med. Entomol..

[142] Gage K.L., Jerrells T.R. (1992). Demonstration and partial characterization of antigens of *Rickettsia rhipicephali* that induce cross-reactive cellular and humoral immune responses to *Rickettsia rickettsii*. Infect. Immun..

[143] Hunter D.J., Torkelson J.L., Bodnar J., Mortazavi B., Laurent T., Deason J., Thephavongsa K., Zhong J. (2015). The *Rickettsia* endosymbiont of *Ixodes pacificus* contains all the genes of de novo folate biosynthesis. PLoS ONE.

[144] Kurtti T.J., Felsheim R.F., Burkhardt N.Y., Oliver J.D., Heu C.C., Munderloh U.G. (2015). *Rickettsia buchneri* sp. nov.; a rickettsial endosymbiont of the blacklegged tick *Ixodes scapularis*. Int. J. Syst. Evol. Microbiol..

[145] Troyo A., Moreira-Soto R.D., Calderon-Arguedas Ó., Mata-Somarribas C., Ortiz-Tello J., Barbieri A.R., Avendaño A., Vargas-Castro L.E., Labruna M.B., Hun L. (2016). Detection of rickettsiae in fleas and ticks from areas of Costa Rica with history of spotted fever group rickettsioses. Ticks Tick Borne Dis..

[146] Metzger M.E., Hardstone Yoshimizu M., Padgett K.A., Hu R., Kramer V.L. (2017). Detection and establishment of *Aedes aegypti* and *Aedes albopictus* (Diptera: Culicidae) mosquitoes in California, 2011–2015. J. Med. Entomol..

